# Complete Genome Sequence of Pseudomonas aeruginosa Strain AA2 (LMG 27630), an Early Isolate Recovered from the Airway of a German Cystic Fibrosis Patient

**DOI:** 10.1128/MRA.00526-20

**Published:** 2020-06-25

**Authors:** Andrea Sass, Tom Coenye

**Affiliations:** a Laboratory of Pharmaceutical Microbiology, Ghent University, Ghent, Belgium; University of Arizona

## Abstract

Pseudomonas aeruginosa is an opportunistic pathogen that is able to cause various infections, including airway infections in cystic fibrosis patients. Here, we present the complete closed and annotated genome sequence of P. aeruginosa AA2, an isolate obtained early during infection of the respiratory tract of a German cystic fibrosis patient.

## ANNOUNCEMENT

Pseudomonas aeruginosa AA2 was retrieved from a German cystic fibrosis patient 6 months after the first P. aeruginosa-positive culture (1–3). Phenotyping revealed that strain AA2 is more virulent than later sequential isolates (AA33 and AA44) from the same patient (2).

Strain AA2 is a member of an international P. aeruginosa reference panel, has multilocus sequence type 708 (4, 5), and was deposited in the BCCM/LMG Bacteria Collection (http://bccm.belspo.be) as LMG 27630. It was previously genome sequenced for the International Pseudomonas aeruginosa Consortium database (6) and for an *in vitro* evolution study in our laboratory (7). In order to further study the adaptation and evolution of strain AA2, including structural variants caused by transposition events, the genome was closed with the aid of PacBio technology.

Cells were grown in liquid culture overnight, inoculated directly from −80°C frozen stock to minimize the number of passages, and then pelleted by centrifugation.

For Illumina sequencing, DNA was extracted using bead beating (7). Libraries were prepared with the NEBNext kit (New England Biolabs) and sequenced on the Illumina NextSeq 500 platform (7). A total of 5,350,348 reads (150 bp) were obtained, 96.9% of which were paired.

For PacBio sequencing, DNA extraction was performed with the Wizard genomic DNA purification kit (Promega). Extracted DNA resulted in one band of >10 kb on a 0.7% agarose gel. DNA was quantified using a Qubit 2.0 fluorometer, and a standard SMRTbell library was prepared with 10-kb insertions. Sequencing was performed on a PacBio Sequel system with SMRTbell template preparation kit 1.0-SPv3 (Pacific Biosciences). Library preparation and PacBio sequencing were performed at Novogene. Original sequencing results were filtered and processed with SMRTlink software, with minLength 0 and minReadScore 0.8 as parameters. A total of 211,898 subreads were obtained, with an average length of 7,857 nucleotides and an *N*_50_ value of 9,252 nucleotides.

Illumina and PacBio reads were assembled with Unicycler v0.4.8 (8), including genome polishing with both short and long reads. Short reads were mapped to the polished assembly to screen for single-nucleotide and structural variants with CLC Workbench v11.0.1 (Qiagen); no variants were detected. Default parameters were used for all software unless stated otherwise.

The P. aeruginosa AA2 genome consisted of a single circular replicon with 6,288,195 bp and a GC content of 66.5%. The Illumina short-read coverage was 126.7×, and the PacBio coverage was 138.1×.

The genome was annotated using the NCBI Prokaryotic Genome Annotation Pipeline (PGAP) (9); 5,727 coding sequences were predicted, including 530 hypothetical proteins (9.3%) and 42 pseudogenes. The frameshift in *mexA* that was detected in this isolate in a previous study (4) was confirmed. PHASTER (10) analysis detected one prophage at nucleotide position 670137 to 702242. The genome also contained 12 rRNAs, 63 tRNAs, 1 transfer-messenger RNA, and 3 other noncoding RNA genes, as well as 8 riboswitches.

### Data availability.

The PacBio reads have been deposited in GenBank under BioProject accession number PRJNA623623. The Illumina reads are part of an Array Express entry with accession number E-MTAB-7331 (source name parent t0). The genome accession number is CP051547.1.
